# Characterization and identification of ubiquitin conjugation sites with E3 ligase recognition specificities

**DOI:** 10.1186/1471-2105-16-S1-S1

**Published:** 2015-01-21

**Authors:** Van-Nui Nguyen, Kai-Yao Huang, Chien-Hsun Huang, Tzu-Hao Chang, Neil Arvin Bretaña, K Robert Lai, Julia Tzu-Ya Weng, Tzong-Yi Lee

**Affiliations:** 1 Department of Computer Science and Engineering, Yuan Ze University, Taoyuan 320, Taiwan; 2 Tao-Yuan Hospital, Ministry of Health & Welfare, Taoyuan 320, Taiwan; 3 Graduate Institute of Biomedical Informatics, Taipei Medical University, Taipei 110, Taiwan; 4 Innovation Center for Big Data and Digital Convergence, Yuan Ze University, Taoyuan 320, Taiwan

**Keywords:** ubiquitin conjugation, ubiquitination, substrate site specificity, maximal dependence decomposition, profile hidden Markov model

## Abstract

**Background:**

In eukaryotes, ubiquitin-conjugation is an important mechanism underlying proteasome-mediated degradation of proteins, and as such, plays an essential role in the regulation of many cellular processes. In the ubiquitin-proteasome pathway, E3 ligases play important roles by recognizing a specific protein substrate and catalyzing the attachment of ubiquitin to a lysine (K) residue. As more and more experimental data on ubiquitin conjugation sites become available, it becomes possible to develop prediction models that can be scaled to big data. However, no development that focuses on the investigation of ubiquitinated substrate specificities has existed. Herein, we present an approach that exploits an iteratively statistical method to identify ubiquitin conjugation sites with substrate site specificities.

**Results:**

In this investigation, totally 6259 experimentally validated ubiquitinated proteins were obtained from dbPTM. After having filtered out homologous fragments with 40% sequence identity, the training data set contained 2658 ubiquitination sites (positive data) and 5532 non-ubiquitinated sites (negative data). Due to the difficulty in characterizing the substrate site specificities of E3 ligases by conventional sequence logo analysis, a recursively statistical method has been applied to obtain significant conserved motifs. The profile hidden Markov model (profile HMM) was adopted to construct the predictive models learned from the identified substrate motifs. A five-fold cross validation was then used to evaluate the predictive model, achieving sensitivity, specificity, and accuracy of 73.07%, 65.46%, and 67.93%, respectively. Additionally, an independent testing set, completely blind to the training data of the predictive model, was used to demonstrate that the proposed method could provide a promising accuracy (76.13%) and outperform other ubiquitination site prediction tool.

**Conclusion:**

A case study demonstrated the effectiveness of the characterized substrate motifs for identifying ubiquitination sites. The proposed method presents a practical means of preliminary analysis and greatly diminishes the total number of potential targets required for further experimental confirmation. This method may help unravel their mechanisms and roles in E3 recognition and ubiquitin-mediated protein degradation.

## Introduction

**Figure 1:**
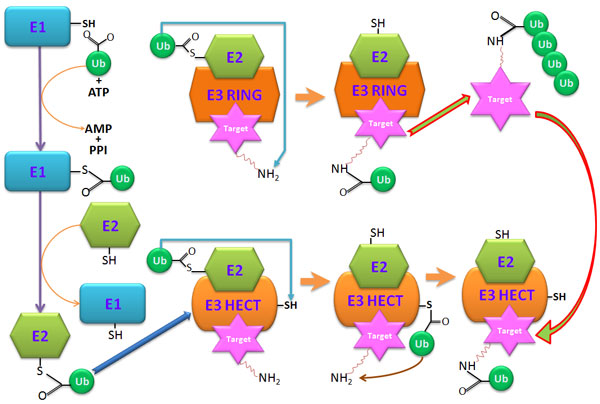
**The reaction process of protein ubiquitination**.

Protein ubiquitination is an essential post-translational modification (PTM) involving the conjugation of an ubiquitin or poly-ubiquitin chains at specific substrate sites [1,2]. In addition to transcriptional regulation, development, apoptosis, and cell proliferation [3], the ubiquitination system is responsible for the selective degradation of several short-lived proteins in eukaryotic cells [4,5]. The ubiquitin-mediated protein degradation is a sequential process involving a group of enzymes known as E1 (activating enzyme), E2 (conjugating enzyme) and E3 (ubiquitin ligase) [5]. As depicted in Figure 1, the ubiquitin-proteasome pathway is a complex and multi-step reaction process. Firstly, ubiquitination is initiated by the attachment of the C-terminal residue of a ubiquitin to a cysteine sulphydryl group in enzyme E1. The ubiquitin is subsequently transferred to E2, which can be conjugated with various E3 enzymes. The ubiquitin ligase E3 recognizes a specific protein substrate and catalyzes the attachment of ubiquitin to the target protein, usually at a lysine (K) residue containing site [5]. Finally, the substrate is sent to the 26S proteasome for degradation. An additional enzyme E4 is responsible for stabilizing and extending the poly-ubiquitin chain [6].

The important role of protein ubiquitination plays in cells has led to an increasing interest in computational identification of ubiquitination sites (Ubi-sites) [7-13]. However, most tools were developed on the basis of small-scale protein data. With the advancement in proteomics technology, it has become necessary to construct new models scalable and practical for big proteome data. Recently, two new approaches had been introduced for identifying Ubi-sites from large-scale proteome data [14,15]. The UbiProber integrated key position and amino acid redisude features specifically designed for large-scale to predict both general and species-specific Ubi-sites [14]. The analysis of UbiProber also showed that: 1) ubiquitination patterns are conserved across different species; 2) some key positions and key amino acid residues are essential for improving the prediction performance; 3) the physicochemical properties of residues in the flanking sequences surrounding a Ubi-site are important in the ubiquitination process. For Ubi-site identification in humans, hCKSAAP_UbSite [15] has utilized amino acid patterns and properties to improve the prediction performance. The area under the receiver operating characteristic (ROC) curve (AUC) was 0.770 and 0.757 for the training and testing data set, respectively.

Although UbiProber and hCKSAAP_UbSite had demonstrated both accuracy and stability, there was room for improvements on the performance. In addition, there was still a lack of Ubi-site identification tools for large-scale data. Consequently, we were motivated to develop a new method to predict Ubi-sites based on their substrate site specificities. The five-fold cross-validation was adopted to evaluate the performance of the predictive models. When applied on the training data set, the model generated an accuracy of 60.17% and MCC of 0.202. On the testing data set, the model obtained an accuracy of 61.30% and MCC of 0.225. In addition, the maximal dependence decomposition (MDD) was employed to improve the predictive performance. The average performance of the model with the integration of MDD was better than that without MDD, reaching an overall 67.93% in accuracy and an MCC value of 0.363. Furthermore, the independent testing also revealed that the combined MDD-models yielded the highest performance. The sensitivity, specificity, accuracy and MCC were 87.76%, 70.02%, 76.13%, and 0.549, respectively. Thus, this would be an important and promising approach for researchers who are interested in identifying ubiquitination sites, especially for large-scale proteome data.

## Materials and method

### Data collection and pre-processing

**Figure 2:**
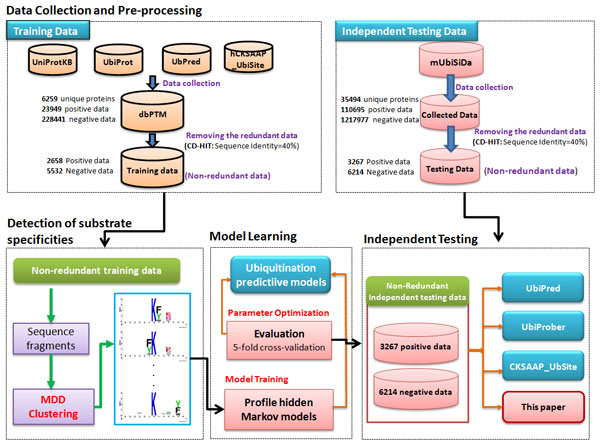
**The system flowchart**.

Figure 2 presents the system flow of this work, consisting of data collection and pre-processing, detection of substrate site specificities, model learning and cross-validation, as well as independent testing. In this work, the training data were mainly collected from dbPTM [16-18], which integrated published literatures and public resources including UniProtKB/Swiss-Prot [19] and UbiProt [20]. As provided in Table 1 totally 6259 experimentally validated ubiquitinated proteins were obtained and used as the training data set. To construct the positive data (Ubi-sites), the window size of 2n + 1 was used to extract fragments containing *n *upstream and *n *downstream flanking residues around the central Lysine (K) residue with the annotation of ubiquitination sites. The negative data (non-Ubi-sites) were constructed by extracting only fragments with the central lysine residue 'K' that has not been verified as ubiquitinated. According to previous works [14] on the performance evaluation of various window lengths, the window size of 13 (n = 6) performed best in the prediction of ubiquitination sites. As a result, the training data set contained 23949 positive and 228441 negative data.

**Table 1:** Data statistics of collected ubiquitination sites.

Resource (data set)	Number of ubiquitinated proteins	Number of ubiquitinated lysines	Number of non-ubiquitinated lysines
**dbPTM **(Training set)	6259	23949	228441

**mUbiSiDa **(Independent testing set)	35494	110695	1217977

To prevent overestimation on the predictive performance, it is necessary to remove homologous fragments from the training data. Following previous studies, the homologous fragments were removed using CD-HIT [21] by repeating these three steps: 1) forming a cluster with a representative fragment having the longest length; 2) Comparing this fragment with the remaining fragments; 3) removing the target fragment if its similarity with the representative fragment is higher than the given threshold, a user-selected value refers to the pairwise sequence identity between two fragments Table 2 shows the results of removing the homologous fragments using CD-HIT based on some values of sequence identity. Moreover, because this study was based on fragments and Ubi-sites, so it is possible that some negative data are identical with some positive data in the training data set, potentially resulting in over-fitting. Therefore, CD-HIT was applied again (by running cd-hit-2d across positive and negative data with 100% sequence identity in the CD-HIT suite) to solve this problem. After having filtered out homologous fragments with 40% sequence identity, the training data set contained 2658 positive and 5532 negative data. In addition, to examine the position-specific amino acid composition for the positive training data, WebLogo [22] was applied to generate the graphical sequence logo for the relative frequency of the corresponding amino acid at each position around ubiquitination sites.

**Table 2:** Data statistics after using CD-HIT.

Sequence identity	Training data set (6259)		Testing data set (35494)	
	
	Positive	Negative	Positive	Negative
100% (original)	23949	228441	110695	1217977

90%	21621	196808	38739	325640

80%	21165	179691	36647	284713

70%	20709	165560	35165	255134

60%	18588	115296	29810	162044

50%	10216	34428	14210	41700

**40%**	**2658**	**5532**	**3267**	**6214**

### Detection of substrate site specificities

With the recent advancements in proteomics technology, more and more experimental data on ubiquitin conjugation sites become available, giving us new opportunities to work on large-scale data. However, the complexity of large-scale data also presents to be a challenge. Although several prediction tools have demonstrated stability and effectiveness, the performance was still required significant improvement. In this work, Maximal Dependence Decomposition (MDD) [23] was applied to detect the substrate site specificities of protein ubiquitination. In our previous works [24-30], we used the MDD approach on amino acid sequences instead of nucleotides, and successfully identified conserved motif and improved the prediction performance. MDD adopts a chi-square *χ*^2^(A_i_, A_j_) test to iteratively assess the dependence of amino acid occurrence between two positions A_i _and A_j _that surround the Ubi-sites. In this study, MDD was applied to sub-divide the positive training data (2658 Ubi-sites fragments) to ten subgroups containing significant substrate motifs. The negative data for each MDD-clustered subgroups were randomly selected from the negative training (5532 non-Ubi-fragments) with a ratio approximately equal to 1:2.08 (same as the ratio of positive training to negative training--5532:2658). As illustrated in Figure S1 (in Additional File 1), these subgroups were used to generate profile hidden Markov models (profile HMMs) for the identification of protein ubiquitination sites with their corresponding substrate motifs.

### Model construction and cross-validation

A public software, HMMER [31], was adopted to generate profile HMMs from the fragment sequences of each MDD-clustered subgroup. An HMM can detect distant relationships between amino acids surrounding the ubiquitination sites. In general, profile HMM learns a predictive model from positive dataset of a class; thus, in this study, only ubiquitinated data (positive training set) was utilized to build a predictive model. For each model of the MDD-clustered subgroups, a threshold parameter is selected as a cut-off value in identifying potential positive data from a query sequence. To search the hits of a HMM, HMMER returns both a bit score and an expectation value (E-value). A search result with an HMMER bit score greater than the threshold parameter is taken as a positive prediction. Prior to the construction of a final model, the predictive performance of the models with varying parameters are evaluated by performing k-fold cross validation. In doing k-fold cross validation, the training data is divided into k groups by splitting each dataset into approximately equal sized subgroups. The advantage of k-fold cross-validation is that all original data are regarded as both training set and test set, and each data is used for testing exactly once [32]. In this study, k is set to five. The models are initially evaluated using five-fold cross-validation and are gauged by measuring their predictive performance. The following measurements were employed to assess the performance of the predictive model: Sensitivity $\text{Sensitivity}\;\left(\text{SEN}\right)=\frac{\text{TP}}{\text{TP}+\text{FN}}$, $\text{Specificity}\;\left(\text{SPE}\right)=\frac{\text{TN}}{\text{TN}+\text{FP}}$, and $\text{Accuracy}=\frac{\text{TP}+\text{TN}}{\text{TP}+\text{FN}+\text{TN}+\text{FP}}$, where TP, TN, FP and FN represent the numbers of True Positives, True Negatives, False Positives and False Negatives, respectively. Mathews Correlation Coefficient (MCC) $\mathrm{Mathews}\;\mathrm{Correlation}\;\mathrm{Coefficient}\;\mathrm{(MCC)}=\frac{\mathrm{TP}\times \text{TN}-\text{FP}\times \mathrm{FN}}{\sqrt{\text{(TP}+FP)(TP+FN)(TN+FP)(\text{TN}+\text{FN})}}$ was also used to access the quality of the predicted result to the observed data. Finally, the models with best predictive performance were further evaluated using the independent testing data.

### Independent testing

Due to the over-fitting problem of the training data set, the predictive performance of the trained models may be overestimated. Therefore, we constructed an independent testing data set to evaluate for the real case. Recently, Chen et al. [33] released a comprehensive database, named mUbiSiDa, for protein ubiquitination sites in mammals. The data set downloaded from mUbiSiDa included 35494 proteins was selected as the independent testing data. The positive and negative data were generated using the same approach as applied to the training data, resulting in 110695 positive and 1217977 negative data. To avoid data redundancy, the homologous fragments were removed using CD-HIT with the sequence identity at 40%. Next, fragments in the negative data that were identical to the positive were filtered out to prevent over-fitting. As a result, the final independent testing data consisted of 3267 positive and 6214 negative data (Table 2). This testing data set was also used on other prediction tools to compare with our models in terms of performance.

## Results and discussion

### Amino acid composition of ubiquitination sites

**Figure 3:**
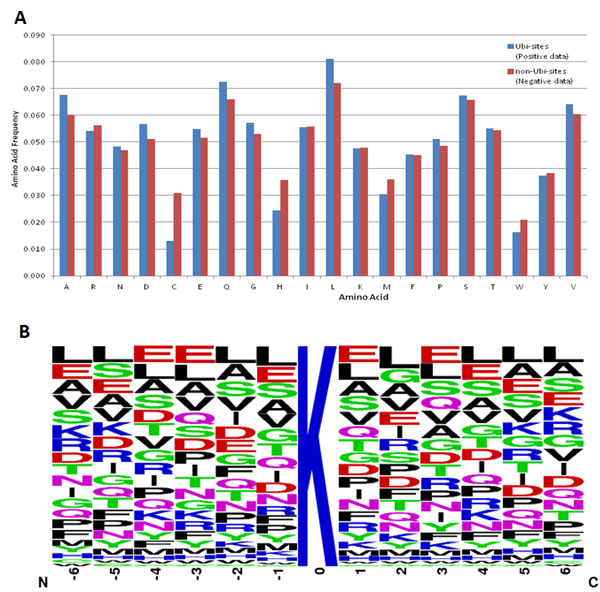
**Composition of amino acids surrounding ubiquitination sites**. (A) Comparison of amino acid composition between Ubi-sites (blue) and non-Ubi-sites(red). (B) Position-specific amino acid composition surrounding Ubi-sites.

The amino acid composition (AAC) could be used to determin the the frequencies of twenty amino acids surrounding the ubiquitination sites in a specified window length. Comparison of the AAC between Ubi-sites and those of non-Ubi-sites provides significant information for the identification of protein ubiquitination sites. As shown in Figure 3A, prominent amino acid residues included Ala (A), Gln (Q), Leu (L), Ser (S), and Val (V), while Cys (C) and Trp (W) were two of the least significant amino acid residues. Additionally, the position-specific amino acid composition surrounding ubiquitination sites (at position 0) could be graphically visualized in a frequency plot of sequence logo [22,23], such that the abundance of amino acids around the substrate sites could be easily observed. As presented in Figure 3B, Sequence logo indicated that lysine (K) residue is the most conserved amino acid residues surrounding the Ubi-sites. It also showed that the most common and prominent amino acid residues included Leu (L), Glu (E), Asp (D), Ala (A), Gln (Q), Ser (S), and Val (V). It has been reported that the composition of flanking amino acids could contribute to the identification of the potential protein modification sites. Thus, in this work, the abundant amino acids could be subsequently used to evaluate their ability to distinguish Ubi-sites from non-Ubi-sites by five-fold cross-validation.

### Substrate motifs of ubiquitination sites

In the detection of substrate motifs of ubiquitination sites, maximal dependence decomposition (MDD) is executed multiple times with varying values in order to obtain the most optimal minimum cluster size. Setting the minimum cluster size to 80 for positive training data yielded nine clusters containing significant motifs as shown in Table 3. The flanking amino acids (-6 ~ +6) of the non-redundant ubiquitination sites, which are centered on position 0, are graphically visualized as an entropy plot of sequence logo. This investigation indicates that the ubiquitinated sequences in each subgroup clustered using MDD give a conserved motif representing its substrate site specificity. As given in Table 3 most of the MDD clusters contain the conserved motifs of Phenylalanine (F), Tyrosine (Y) and Trytophan (W) residues. For example, MDD cluster 1 gives a conserved motif of Phenylalanine (F), Tyrosine (Y) and Trytophan (W) residues at position +1. Similarly, MDD cluster 2 was comprised of a conserved motif of Phenylalanine (F), Tyrosine (Y) and Trytophan (W) residues at position -1. In general, almost all clusters containing conserved Phenylalanine (F), Tyrosine (Y) and Trytophan (W) at specific positions. This suggests that, for ubiquitination, the substrate site specificities may depend on the conserved position of Phenylalanine (F), Tyrosine (Y) and Trytophan (W) residues.

**Table 3:** MDD-identified substate motifs for 2658 Ubi-sites (positive training data).

MDD Group	Number of Ubi-sites	Sequence Logo
1	98	

2	87	

3	191	

4	574	

5	423	

6	131	

7	122	

8	260	

9	75	

10	697	

### Cross-validation performance

A profile HMM was trained will all positive training data to examine the effectiveness of AAC in identifying Ubi-sites. As shown in Table 4 the single HMM yielded 61.60% sensitivity, 57.20% specificity, 58.26% accuracy, and a MCC value of 0.175, based on five-fold cross-validation evaluation. With an attempt to enhance the practicability and stability of our model on large-scale data, the MDD approach was applied for the improvement of prediction performance. For instance, MDD cluster 1, which contains a conserved motif of Phenylalanine (F), Tyrosine (Y) and Trytophan (W) residues at position +1, obtained 77.55% sensitivity, 70.59% specificity, 72.85% accuracy, and a MCC value of 0.453. Similarly, MDD cluster 2 also yielded a significant improvement with a sensitivity of 94.32%, a specificity of 85.56%, an accuracy of 88.43%, and a MCC value of 0.764. In general, almost all clusters containing conserved Phenylalanine (F), Tyrosine (Y) and Trytophan (W) at specific positions, could yield better prediction performance. In contrast, other clusters without clearly conserved motifs containing Phenylalanine (F), Tyrosine (Y) and Trytophan (W) residues generally showed lower sensitivity and accuracy (≤70%). The results of five-fold cross-validation indicate that most of the MDD-models outperformed single HMM. The average performance of MDD-clustered HMMs was 67.93% in accuracy, with an MCC value of 0.363. After applying MDD, the improvement on accuracy and MCC was 9.67% (from 58.26% to 67.93%) and 0.188 (from 0.175 to 0.363), respectively. This investigation demonstrated the effectiveness of the MDD-identified substrate motifs in the prediction of protein ubiquitination sites. Then, the independent testing data were subsequently employed to evaluate the effectiveness of the MDD-clustered HMMs against the non-MDD model (single HMM).

**Table 4:** Performance evaluation by five-fold cross-validation for all data and 10 MDD-clustered subgroups.

Data set	SEN	SPE	ACC	MCC
**All data**	**62.60%**	**59.00%**	**60.17%**	**0.202**

**MDD cluster 1**	77.55%	70.59%	72.85%	0.453

**MDD cluster 2**	94.32%	85.56%	88.43%	0.764

**MDD cluster 3**	84.82%	70.53%	75.17%	0.519

**MDD cluster 4**	60.80%	58.24%	59.07%	0.178

**MDD cluster 5**	66.90%	60.68%	62.70%	0.258

**MDD cluster 6**	72.52%	68.13%	69.55%	0.382

**MDD cluster 7**	70.49%	62.60%	65.16%	0.310

**MDD cluster 8**	62.31%	54.90%	57.30%	0.161

**MDD cluster 9**	70.67%	58.97%	62.77%	0.278

**MDD cluster 10**	70.30%	64.44%	66.34%	0.326

**10 MDD clusters (average)**	**73.07%**	**65.46%**	**67.93%**	**0.3629**

### Independent testing performance

**Figure 4:**
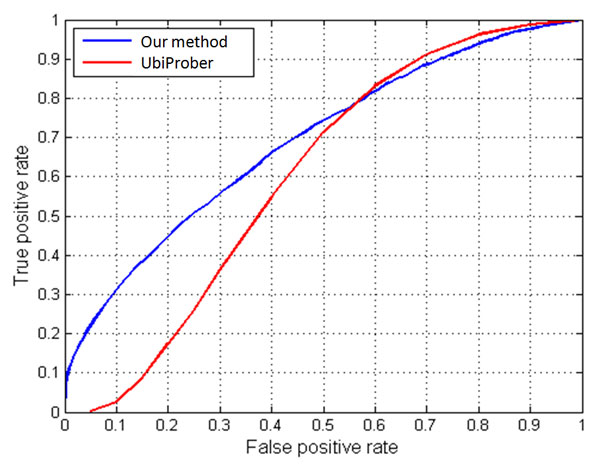
**The comparison of ROC curve between our proposed method and UbiProber**.

As mentioned previously, to assess the practicability of the trained models, an independent testing data set was constructed from mUbiSiDa. After data pre-processing, the independent testing data set comprised 3267 positive and 6214 negative data. The performance of the model when tested on the independent testing data set was shown in Table 5. The single HMM trained with all positive training data gives 63.54% sensitivity, 60.12% specificity, 61.30% accuracy, and 0.225 MCC value for independent testing data. The independent testing result for each MDD group was also given in Table 5. The combination of ten MDD models (MDD-clustered HMMs) achieved the highest performance with 87.76% sensitivity, 70.02% specificity, 76.13% accuracy, and the MCC value of 0.549. Additionally, our proposed model was also compared with an existing prediction tool, UbiProber [14], using the same independent testing data set. It has integrated key position and amino acid redisude features specifically designed for large-scale to predict both general and species-specific Ubi-sites. Figure 4 provides the comparison of ROC curve between our proposed method and UbiProber and indicates that our method achieved a slightly higher AUC (62.43%) than UbiProber (62.06%).

**Table 5:** Independent testing performance for single HMM and MDD-clustered HMMs.

Models	SEN	SPE	ACC	MCC
**Single HMM (all data)**	**63.54%**	**60.12%**	**61.30%**	**0.225**

MDD-Model 1	70.16%	66.61%	67.83%	0.351

MDD-Model 2	87.70%	70.47%	76.41%	0.553

MDD-Model 3	74.78%	67.70%	70.14%	0.405

MDD-Model 4	63.27%	57.35%	59.39%	0.196

MDD-Model 5	52.74%	62.13%	58.90%	0.143

MDD-Model 6	77.50%	70.12%	72.66%	0.454

MDD-Model 7	71.66%	60.22%	64.16%	0.303

MDD-Model 8	65.17%	60.57%	62.16%	0.245

MDD-Model 9	68.07%	59.14%	62.22%	0.259

MDD-Model 10	70.65%	67.14%	68.35%	0.360

**MDD-clustered HMMs**	**87.76%**	**70.02%**	**76.13%**	**0.549**

**Figure 5:**
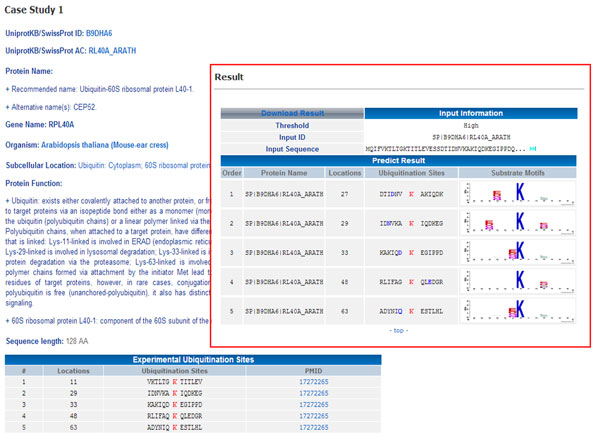
**A case study of the ubiquitination site prediction on *Ubiquitin-60S ribosomal protein L40-1 *(RPL40A) of *Arabidopsis thaliana***.

A case study of ubiquitination site prediction on *Ubiquitin-60S ribosomal protein L40-1 *(RPL40A) of *Arabidopsis thaliana *is given in Figure 5. The RPL40A has five experimentally validated Ubi-sites located at positions 11, 29, 33, 48 and 63 [19,34]. The function(s) of this protein provided by UniProt http://www.uniprot.org/uniprot/RL40A_ARATH showed that Ubiquitin exists either covalently attached to another protein, or free (unanchored). When covalently bound, it is conjugated to target proteins via an isopeptide bond either as a monomer (monoubiquitin), a polymer linked via different Lys residues of the ubiquitin (polyubiquitin chains) or a linear polymer linked via the initiator Met of the ubiquitin (linear polyubiquitin chains). Polyubiquitin chains, when attached to a target protein, have different functions depending on the Lys residue of the ubiquitin that is linked: Lys-11-linked is involved in ERAD (endoplasmic reticulum-associated degradation) and in cell-cycle regulation; Lys-29-linked is involved in lysosomal degradation; Lys-33-linked is involved in kinase modification; Lys-48-linked is involved in protein degradation via the proteasome; Lys-63-linked is involved in endocytosis, and DNA-damage responses. As shown in Figure 5, the proposed model predicted five ubiquitination sites in the query sequence with positions 27, 29, 33, 48 and 63. The position 27 is a false positive prediction and the accuracy reaches 80%.

### Interactions between E3 ligases and ubiquitinated proteins

As mentioned previously, the ubiquitin-mediated protein degradation is a sequential process involving in 3 major kinds of enzymes: activating enzyme E1, conjugating enzyme E2 and ubiquitin ligase E3. In the ubiquitin-proteasome pathway, E3 ligases play very important roles by recognizing a specific protein substrate and catalyzing the attachment of ubiquitin to the target protein, usually at a lysine (K) residue containing site. Therefore, a full understanding about the interactions between E3 ligases and ubiquitination substrate proteins has been being an emerging study in the investigation of protein ubiquitination regulatory network. In order to provide a further investigation for the interactions between E3 ligases and ubiquitinated proteins, we have collected E3 ligases and their protein-protein interactions in human and mouse. The experimentally verified E3 ligases were collected from four resources, as shown in Table S1 (in Additional File 1). After the removal of data redundancy, the non-redundant data of E3 ligases contained 501 entries in human and 232 entries in mouse. The ubiquitination substrate (Ubi-substrate) proteins were extracted from Ubi-substrate training and independent testing data sets which were used for training and testing predictor in identification of ubiquitination sites mentioned previously. As a result, 32260 Ubi-substrate proteins on Human and 5195 Ubi-substrate proteins on Mouse were obtained. In addition, basing on protein-protein interaction, the relationships between E3 ligases and Ubi-substrate proteins were investigated. Through the investigation of protein-protein interactions, Table S2 (in Additional File 1) shows that the 501 human and 232 mouse E3 ligases interact with 3938 human and 604 mouse ubiquitinated proteins along with 17397 human and 2949 mouse ubiquitination site, respectively.

## Conclusion

The recent rapid accumulation of proteomics data has given us the opportunity to mine large amounts of protein data, extract important information about ubiquitination, and build models to identify ubiquitination sites. However, the performance of existing Ubi-site identification tools still appear to be hampered when dealing with large data sets as our experimentation has suggested. Thus, it is necessary to develop an effective approach to improve the efficiency of prediction. Previously, we have demonstrated the ability of MDD to enhance the performance of predictive models by clustering a large set of aligned signal sequences into subgroups [23,35-37]. In this work, we applied the similar strategy combined with profile hidden Markov models in a prediction model for Ubi-site identification and obtained an overall improvement in all performance measures. Additionally, evaluation of our model with an independent testing data set showed the strength of our approach in comparison to an existing prediction tool. To further enhance the performance of our model for practical applications on large-scale data, we applied MDD to sub-divide the positive training data into subgroups with statistically significant information. Therefore, this work has demonstrated that the MDD-clustered HMMs could provide promising predictive ability in identifying ubiquitination sites from large-scale proteome data.

## Competing interests

The authors declare that they have no competing interests.

## Authors' contributions

JTYW and TYL conceived and supervised the project. VNN, KYH, CHH, THC and NAB were responsible for the design, computational analyses, implemented the experiments, and drafted the manuscript with revisions provided by KRL, JTYW, and TYL. All authors read and approved the final manuscript.

## Supplementary Material

Additional File 1 **Supplementary Tables and Figures**. Contains additional Tables and Figures showing further results in this study. Click here for file

## Declarations

Publication charge for this work was funded by MOST grant 103-2221-E-155-020-MY3 to TYL.

This article has been published as part of *BMC Bioinformatics *Volume 16 Supplement 1, 2015: Selected articles from the Thirteenth Asia Pacific Bioinformatics Conference (APBC 2015): Bioinformatics. The full contents of the supplement are available online at http://www.biomedcentral.com/bmcbioinformatics/supplements/16/S1
