# Supplementary material for: Characterization and identification of ubiquitin conjugation sites with E3 ligase recognition specificities
Source: BMC Bioinformatics. 2015 Jan 21;16(Suppl 1):S1. doi: 10.1186/1471-2105-16-S1-S1 (PMC4331700; doi:10.1186/1471-2105-16-S1-S1)
Supplement: Additional File 1 — Supplementary Tables and Figures. Contains additional Tables and Figures showing further results in this study. [file 1471-2105-16-S1-S1-S1.docx]

**Supplementary materials**

**
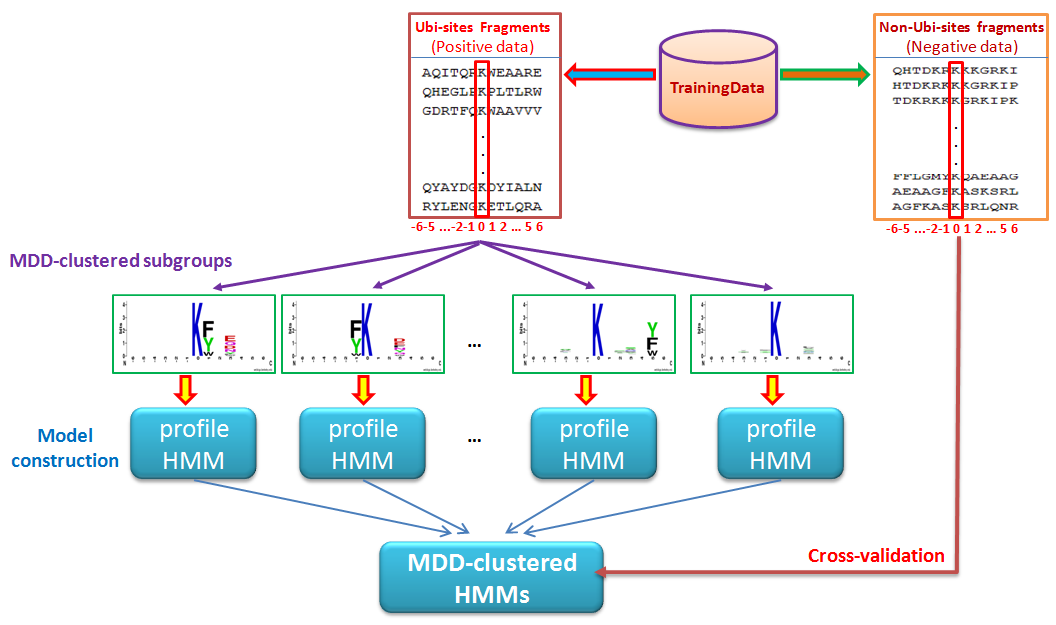
**

**Figure S1. The conceptual flowchart of constructing the MDD-clustered HMMs.**

**Table S1. Data statics of the collected ubiquitin ligases (E3)**

| **Resource** | **Human** | **Mouse** | **Total** |
| --- | --- | --- | --- |
| UniProt-27.6.14 | **208** | **199** | **407** |
| E3NET | **101** | **66** | **167** |
| hUbiquitome | **136** | **0** | **136** |
| UUCD-V1 | **475** | **71** | **546** |
| **Combined non-redundant data** | **501** | **232** | **733** |

**Table S2. Data statistics for E3 ligases, ubiquitinated proteins, and their interactions.**

| **Species** | **Human** | **Mouse** | **Total** |
| --- | --- | --- | --- |
| **Number of E3 ligases** | 501 | 232 | 733 |
| **Number of ubiquitinated proteins** | 32260 | 5195 | 37455 |
| **Number of Ubi-sites** | 98096 | 19721 | 117817 |
| **Number of interactions between E3 ligases and ubiquitinated proteins** | 41942 | 3236 | 45178 |
| **Number of proteins interacting with E3 ligases** | 8077 | 1977 | 10054 |
| **Number of Ubi-proteins interacting with E3 ligases** | 3938 | 604 | 4542 |
| **Number of Ubi-sites in Ubi-proteins interacting with E3 ligases** | 17397 | 2949 | 20346 |
